# Thoracic dysfunction in whiplash-associated disorders: a systematic review and meta-analysis protocol

**DOI:** 10.1186/s13643-016-0201-0

**Published:** 2016-02-09

**Authors:** Nicola R Heneghan, Richard Smith, Alison Rushton

**Affiliations:** School of Sport, Exercise and Rehabilitation Sciences, College of Life and Environmental Sciences, University of Birmingham, Edgbaston, Birmingham, B15 2TT UK

**Keywords:** Thoracic spine, Thoracic dysfunction, Whiplash associated disorder, Systematic review

## Abstract

**Background:**

Whiplash-associated disorder (WAD) research has largely focused on the neck, yet symptoms often include other areas. The prevalence of acute thoracic spine pain is reported ~66 %, which is perhaps unsurprising given the mechanism of injury involves a forceful loading/eccentric contraction of posterior thoracic structures such as the trapezius. Many individuals with WAD experience disability and pain beyond normal tissue healing time, termed chronic WAD. With the thoracic spine contributing to neck mobility, and 23 % of individuals complaining of thoracic pain 1 year post injury, it is time to look beyond the neck to fully understand the anatomical dysfunction in WAD.

**Methods/Design:**

A systematic review protocol has been designed and will be reported in line with Preferred Reporting Items for Systematic Reviews and Meta-Analyses Protocols (PRISMA-P). A sensitive topic-based search strategy is planned from inception to the current date. Databases, grey literature and registers will be searched using terms and keywords derived from a scoping search. Two reviewers will independently search information sources, assess studies for inclusion and extract data. A third reviewer will check for accuracy. Data to be extracted include summary data: sample size and characteristics, timescales to reflect disorder state, patient-reported or performance-based measure and findings. Risk of bias within studies will be assessed using the Newcastle-Ottawa Scale. Quantitative meta-analysis approach will be used for homogenous data and where appropriate presented using subgroups. All other results will be presented using narrative summaries. Subgroups will, where possible, be based on patient-reported or performance-based measure of dysfunction and/or stage of condition (acute/sub-acute or chronic). Strength of the overall body of evidence will be assessed using Grading of Recommendations Assessment, Development and Evaluation (GRADE).

**Discussion:**

This is the first study to bring together evidence of thoracic dysfunction post whiplash and provide new insights into the scope and nature of thoracic dysfunction in WAD. With current management options being largely focused to a primary neck complaint and many patients going to become chronic in their presentations, this review may stimulate research and clinical interest in a largely under investigated, yet anatomically and kinematically related, spinal region.

**Systematic review registration:**

PROSPERO CRD42015026983.

**Electronic supplementary material:**

The online version of this article (doi:10.1186/s13643-016-0201-0) contains supplementary material, which is available to authorized users.

## Background

The cumulative incidence of patients seeking healthcare post whiplash from a road traffic accident has increased during the last 30 years to an annual incidence of between 3 and 6/1000 inhabitants in North America and Western Europe [1]. Following injury, individuals experience a range of clinical manifestations, described as whiplash-associated disorder (WAD), including neck pain, fatigue, nausea, low self-reported physical and mental health, cognitive problems and pain in multiple sites [2]. The severity of presentation in WAD is categorised according to Quebec Task Force (QTF) Classification where the presence of clinical signs and symptoms relate to severity of the disorder [3].

Whilst research has identified risk factors for poor prognosis [4, 5], and enhanced understanding of neurophysiological changes [6], we do not know why disability and pain persist beyond the normal tissue healing times. With 40–60 % patients progressing to experience chronic whiplash associated disorder (CWAD) and estimated costs of $3.9 billion (USA) and €10 billion (Europe) associated with management and time off work [7, 8], further research is needed to better understand the impact of this trauma on anatomically related body regions, such as the thoracic spine. This may perhaps explain why there is inconclusive evidence for the effectiveness of physiotherapy management for whiplash-associated disorder II, where interventions targeted a primary complaint of neck pain [9]. CWAD research has focused on the primary complaint of neck pain [10] although symptoms may also include stiffness [11, 12] and pain in other regions including the jaw, head, upper and lower limbs, chest, abdomen and groin [13]. Data from a large cohort study (*n* = 6481) reported that 66 % of individuals complained of mid-spine pain post whiplash injury, with 23 % still experiencing symptoms 1 year on [13]. This is unsurprising given the mechanism of injury involving forceful stretch loading to the upper back muscles, which span both the neck and mid-spine [14]. Evidence of trapezius muscle abnormalities are well documented in CWAD [15, 16], with recent evidence of pathology in the mid/lower portion of the muscle where it inserts onto the bone (myofascial-entheseal dysfunction) [17]. This may partly account for the prevalence of 65.5 % (95 % CI 64.4–66.7) thoracic pain in acute WAD [13].

The thoracic spine contributes up to 33 and 21 % of the movement occurring during neck flexion and rotation, respectively [18]; however, little is known about the impact of WAD on this spinal region [19]. Further research is needed to examine the impact of injury on the thoracic spine in WAD. Knowledge of such dysfunction may be used to inform clinical examination of patients with WAD and future clinical trials of novel interventions targeting thoracic impairments in WAD. In the first instance, a systematic review of the current evidence is needed to examine the scope and nature of dysfunction/impairment in the thoracic spine region following WAD.

### Objectives

The aim of this review is to synthesise the evidence of thoracic dysfunction in patients with WAD. A secondary aim is to explore the scope and nature of such changes based on severity using the QTF and post injury stage, acute/sub-acute (up to 3 months) or chronic (>6 months).

## Methods/design

### Protocol and registration

The design and methods used to inform this systematic review protocol comply with the Centre of Research and Dissemination Guidelines [20] and Meta-analyses of Observational Studies in Epidemiology (MOOSE) guidelines [21] and is reported in line with the Preferred Reporting Items for Systematic Reviews and Meta-Analyses Protocols (PRISMA-P) ([22], see Additional file 1). Eligibility criteria were informed using the Sample, Phenomenon of Interest, Design, Evaluation, and Research (SPIDER) and MOOSE guidelines. The sample (S) comprised adults patients aged >19 years. The phenomenon of interest (PI) is a whiplash-associated disorder following motor vehicle or sporting injury. All types of observational study design (D) will be considered, case control, cohort and single case studies. Any patient-reported or performance-based measure of thoracic dysfunction will be evaluated (E). Included research types (R) will be quantitative research. The protocol is registered with the PROSPERO (Registration number: CRD42015026983).

### Information sources

The search will employ sensitive topic-based strategies designed for each database from inception to November 25, 2015. There will be no language or geographical restrictions. Databases will include Cumulative Index to Nursing and Allied Health Literature (CINAHL), Excerpta Medica Database (EMBASE), Medical Literature Analysis and Retrieval System Online (MEDLINE), Zetoc, Index to Chiropractic Literature ChiroAccess and Google Scholar. Selected Internet sites and indexes will be used including Turning Research into Practice and PubMed. The National Research Register and Cochrane Back Review Group will be searched. Hand searching of key journals will include Spine and European Spine Journal. Grey literature searching will include British National Bibliography for Report Literature, Dissertation Abstracts, Index to Scientific and Technical Proceedings, National Technical Information Service and the System for Information on Grey Literature.

### Search strategy

The search strategy will include the phenomenon of interest and patient-reported or performance-based measure of thoracic dysfunction. Terms and keywords derived from scoping search and expertise (subject specific and methodological) in the subject field will include the following: ‘whiplash’, ‘whiplash associated disorder’, ‘WAD’, ‘whiplash injury’, ‘motor vehicle accident OR collision’, ‘road traffic accident’, ‘cervical strain’ and ‘thoracic spine’, ‘dorsal spine’, ‘mid-spine’ and ‘thoracic injuries’, limiting to adults >19 years and diagnosis to achieve best balance of sensitivity and specificity. Example of search from MEDLINE is included (see Additional file 2). Terms will be adapted to reflect differences in spellings and unique searching features of individual databases. Articles must be peer reviewed, and reference lists for included papers will be searched.

### Study records

#### Data management

Records will be managed through EndNote, specific software for managing bibliographies.

### Selection process

Two reviewers will search information sources independently and assess identified studies for inclusion, facilitated by grading each eligibility criterion as eligible/not eligible/might be eligible [23]. The full text of a study will be reviewed, and a study is considered potentially relevant when it cannot be clearly excluded on the basis of its title and abstract [20] following discussion between the two independent reviewers. Full text will be obtained for abstracts with insufficient information or in a situation of disagreement. A study will be included when both reviewers independently assess it as satisfying the inclusion criteria from the full text. A third reviewer will mediate in the event of disagreement following discussion [24].

### Data collection process and items

Using a standardised form, two reviewers will extract the data independently [20]. A third reviewer will independently check the data for consistency and clarity. Data extracted will include the following summary data: sample characteristics, sample size, outcomes of interest, and timescales to reflect disorder state, acute/sub-acute and chronic, outcomes.

### Risk of bias in individual studies

Risk of bias for each included trial will be independently assessed by the same initial reviewers. The third reviewer will mediate in situations of disagreement. Cohen’s *κ* will be used to assess agreement between reviewers. All tools and processes will be piloted prior to use. Risk of bias will be assessed using the Newcastle-Ottawa Scale [25] where eight items are rated and categorised into three groups for cohort studies or case control studies, namely selection, comparability and outcome. For cohort studies, the items include (1) the selection of the study groups (representativeness of the exposed cohort, selection of non-exposed controls, ascertainment of exposure and demonstration that outcome of interest was not present at start of study), (2) the comparability of the groups based on design or analysis, and (3) outcome (the assessment of outcome, length and adequacy of follow up of cohorts). For case control studies, the items include (1) the selection of the study groups (case definition, representativeness of cases, selection of controls, definition of controls), (2) the comparability of the groups, and (3) exposure (the ascertainment of the exposure or outcome of interest for case-control or cohort studies, respectively, and non-response rate) [25].

### Data

Data of study characteristics will be presented in a tabulated form to include details of the study setting and sample; characteristics of the sample include age; gender; where provided, severity of WAD using the QTF Classification; time point post injury; and patient-reported or performance-based measure of thoracic dysfunction. Authors will be contacted to request raw data (mean, standard deviation and confidence intervals) for patient-reported or performance-based measures or additional data where required. All results will be reported in the context of overall study quality.

### Data synthesis

Transparency in the process of searching and numbers of papers included/excluded will be reported on a PRISMA flow chart. Based on a preliminary scoping search, it is anticipated that clinical heterogeneity will be evident across samples with respect to severity of presentation, time post injury, and evaluated patient-reported or performance-based measure of thoracic dysfunction. The following sections summarise planned processes for data synthesis based on meta-analysis and narrative synthesis.

#### Meta-analysis

Meta-analyses will be conducted for studies with consistent characteristics for:

- Study design, e.g. case control vs cohort
- Measure of thoracic dysfunction, e.g. joint range of motion
- Severity of presentation based on QTF classification, e.g. WAD II
- Stage post injury, e.g. acute, sub-acute or chronic.

Meta-analyses will be performed should a sufficient number of studies are found that share all of the characteristics listed above. There are minimal differences between the studies in other characteristics, e.g. sample, and data for each study is available and reported with sufficient detail [20].

Where the above conditions are met, heterogeneity will be assessed using the *I*^2^ statistic. The interpretation of the *I*^2^ value will be based on the guidelines in the Cochrane Handbook for Systematic Reviews of Interventions, where 0 to 40 % is low, 30 to 60 % is moderate, 50 to 90 % is substantial and 75 to 100 % is considerable heterogeneity [26]. The *P* value from the chi-squared test will also be taken into consideration, with significant heterogeneity being defined with a *P* value <0.10.

Groups of studies in which heterogeneity is found to be low (*I*^2^ < 50 %) will be assessed using meta-analysis. The mean differences (MD) in the dysfunction between groups with and without WAD for different degrees of severe and time points post injury will be combined into summary estimates. Only adjusted values extracted from the primary studies will be used. A random-effects model will be implemented, to account for within-study and between-study variability. Sensitivity analysis will be conducted based on risk of bias; studies with a score of 0 on the NOS will be excluded to determine whether the summary estimate remains the same.

#### Narrative synthesis

In the event the meta-analysis is not appropriate, a narrative synthesis will be performed. Synthesis will bring together evidence of measures of thoracic dysfunction, e.g. thoracic outlet syndrome, muscle dysfunction/pathology and mobility. For each measure of dysfunction, tables will be provided to include summary results and illustrate quality of individual studies using ‘stars’ to reflect quality within the categories of selection, comparability and outcome or exposure, and a statement of results based on outcome and in context to quality (see Additional file 3). Further discussion of findings will be provided in a narrative form within the “Results/discussion” section where results will be contextualised based on the quality of evidence.

### Meta-bias

To assess for publication bias, we will search grey literature and also conference proceedings for national and international conferences in the past 2 years to identify related and unpublished studies. The effect of including grey literature or unpublished studies on outcomes and linked to bias will be reported narratively in the results. Selective reporting of outcomes in all the eligible studies will be assessed to determine whether outcomes that were planned were actually reported in the published studies using protocols where available.

### Confidence in cumulative evidence

The strength of the overall body of evidence will be assessed using Grading of Recommendations, Assessment, Development and Evaluation (GRADE) [27]. Although by their very nature observational studies are considered ‘low quality’, they may be *upgraded* to ‘moderate’ or ‘high’ where a large dose response is evident or the effects cannot be accounted for by bias, something that is common to observational studies [27]. Confidence in cumulative evidence will therefore be rated as ‘high’, ‘moderate’, ’low’ or ‘very low’, where high quality evidence would conclude no further research is likely to have an important impact in confidence of findings, moderate would suggest further research is likely to have an important impact, low would indicate further research is required to have an impact on confidence and very low would indicate significant uncertainty of any effect [27].

## Discussion

Current clinical assessment and management of patients with WAD does not extend to include the thoracic spine, despite evidence of pain and reported dysfunction in the thoracic region. With the current management options being largely focused to a primary neck complaint and many patients going to become chronic in their presentations, this review may stimulate research and clinical interest in a largely under investigated, yet anatomically and kinematically related, spinal region. The strength of this review is that it will bring together evidence of thoracic dysfunction in a range of musculoskeletal structures post whiplash. Furthermore, the reviewers will group findings, where possible, into different stages post injury and different degrees of severity as part of the synthesis. This evidence may then be used to inform guideline development in the management of WAD and planning of further research or to prompt clinicians to consider the involvement and examine the thoracic spine and associated structures within their clinical examination of patients presenting post injury.

### Limitations

We expect our search will yield a spectrum of heterogeneous patient and performance-based measures across a number of post injury periods, acute/sub-acute and chronic. Likewise the measures of dysfunction used may vary across studies and established validity and reliability of such tools in this population may be unknown.

### Dissemination

The results of this systematic review and meta-analysis will be published in peer-reviewed journals and presented at conference where appropriate.

## Additional files

Additional file 1:
**PRISMA-P (Preferred Reporting Items for Systematic review and Meta-Analysis Protocols) 2015 checklist.** (DOC 84 kb) Additional file 2:
**Medline OvidSP advanced search: 1948-25th November 2015.** (DOCX 16 kb) Additional file 3:
**Example of results/summary table.** (DOCX 20 kb)

## Abbreviations

CINAHL: Cumulative Index to Nursing and Allied Health Literature
CRD: Centre for Reviews and Dissemination
EMBASE: Excerpta Medica Database
GRADE: Grading of Recommendations Assessment, Development and Evaluation
MEDLINE: Medical Literature Analysis and Retrieval System Online
MOOSE: Meta-analysis Of Observational Studies in Epidemiology
PRISMA: Preferred Reporting Items for Systematic Reviews and Meta-Analyses
QTF: Québec Task Force; classification system for whiplash associated disorders
SPIDER: Sample, Phenomenon of Interest, Design, Evaluation, Research type
WAD: whiplash-associated disorders
Zetoc: research database
