# Supplementary material for: Thoracic dysfunction in whiplash-associated disorders: a systematic review and meta-analysis protocol
Source: Syst Rev. 2016 Feb 9;5:26. doi: 10.1186/s13643-016-0201-0 (PMC4748634; doi:10.1186/s13643-016-0201-0)
Supplement: Additional file 1: — PRISMA-P (Preferred Reporting Items for Systematic review and Meta-Analysis Protocols) 2015 checklist. (DOC 84 kb) [file 13643_2016_201_MOESM1_ESM.doc]

**Additional File 1.**

**PRISMA-P (Preferred Reporting Items for Systematic review and Meta-Analysis Protocols) 2015 checklist: recommended items to address in a systematic review protocol**

| Section and topic | Item No | Checklist item | Signpost |
| --- | --- | --- | --- |
| ADMINISTRATIVE INFORMATION | | |  |
| Title: |  |  |  |
| Identification | 1a | Identify the report as a protocol of a systematic review | P1 Thoracic dysfunction in whiplash associated disorders: a systematic review and meta-analysis protocol |
| Update | 1b | If the protocol is for an update of a previous systematic review, identify as such | Not applicable |
| Registration | 2 | If registered, provide the name of the registry (such as PROSPERO) and registration number | P2 PROSPERO: CRD42015026983 |
| Authors: |  |  |  |
| Contact | 3a | Provide name, institutional affiliation, e-mail address of all protocol authors; provide physical mailing address of corresponding author | P1 Dr Nicola Heneghan (Corresponding author)  Lecturer in Physiotherapy  School of Sport, Exercise and Rehabilitation Sciences  College of Life and Environmental Sciences  University of Birmingham  Edgbaston, Birmingham,  B15 2TT, UK  Tel: 0121 415 8367  Email: [n.heneghan@bham.ac.uk](mailto:n.heneghan@bham.ac.uk)  Richard Smith  University of Birmingham  [SmithRMW@adf.bham.ac.uk](mailto:SmithRMW@adf.bham.ac.uk)  Dr Alison Rushton  University of Birmingham  [a.b.rushton@bham.ac.uk](mailto:a.b.rushton@bham.ac.uk) |
| Contributions | 3b | Describe contributions of protocol authors and identify the guarantor of the review | P6. NH is CI leading protocol development, analyses and dissemination. RS and NH are first and second reviewers. AR is third reviewer. All authors have contributed to the design and development of the protocol and will contribute to data interpretation and manuscript draft. All authors have approved this final manuscript. |
| Amendments | 4 | If the protocol represents an amendment of a previously completed or published protocol, identify as such and list changes; otherwise, state plan for documenting important protocol amendments | Not applicable |
| Support: |  |  |  |
| Sources | 5a | Indicate sources of financial or other support for the review | P6 ‘No funding was provided to support this study’ |
| Sponsor | 5b | Provide name for the review funder and/or sponsor | Not applicable |
| Role of sponsor or funder | 5c | Describe roles of funder(s), sponsor(s), and/or institution(s), if any, in developing the protocol | Not applicable |
| INTRODUCTION | | |  |
| Rationale | 6 | Describe the rationale for the review in the context of what is already known | P3 Background |
| Objectives | 7 | Provide an explicit statement of the question(s) the review will address with reference to participants, interventions, comparators, and outcomes (PICO) | P3 and P4 |
| METHODS | | |  |
| Eligibility criteria | 8 | Specify the study characteristics (such as PICO, study design, setting, time frame) and report characteristics (such as years considered, language, publication status) to be used as criteria for eligibility for the review | P4 Methods/design |
| Information sources | 9 | Describe all intended information sources (such as electronic databases, contact with study authors, trial registers or other grey literature sources) with planned dates of coverage | P4 Information sources and Search strategy |
| Search strategy | 10 | Present draft of search strategy to be used for at least one electronic database, including planned limits, such that it could be repeated | See additional file 2 |
| Study records: |  |  |  |
| Data management | 11a | Describe the mechanism(s) that will be used to manage records and data throughout the review | P4 Data management |
| Selection process | 11b | State the process that will be used for selecting studies (such as two independent reviewers) through each phase of the review (that is, screening, eligibility and inclusion in meta-analysis) | P4 Selection process |
| Data collection process | 11c | Describe planned method of extracting data from reports (such as piloting forms, done independently, in duplicate), any processes for obtaining and confirming data from investigators | P4 Data collection process and items |
| Data items | 12 | List and define all variables for which data will be sought (such as PICO items, funding sources), any pre-planned data assumptions and simplifications | P4 Data collection process and items |
| Outcomes and prioritization | 13 | List and define all outcomes for which data will be sought, including prioritization of main and additional outcomes, with rationale | P4 Data collection process and items |
| Risk of bias in individual studies | 14 | Describe anticipated methods for assessing risk of bias of individual studies, including whether this will be done at the outcome or study level, or both; state how this information will be used in data synthesis | P4 Risk of bias in individual studies |
| Data synthesis | 15a | Describe criteria under which study data will be quantitatively synthesised | P5 Data synthesis section |
|  | 15b | If data are appropriate for quantitative synthesis, describe planned summary measures, methods of handling data and methods of combining data from studies, including any planned exploration of consistency (such as I^2^, Kendall’s τ) | P5 Data synthesis section |
|  | 15c | Describe any proposed additional analyses (such as sensitivity or subgroup analyses, meta-regression) | P5 Data synthesis section |
|  | 15d | If quantitative synthesis is not appropriate, describe the type of summary planned | P5 Data synthesis section |
| Meta-bias(es) | 16 | Specify any planned assessment of meta-bias(es) (such as publication bias across studies, selective reporting within studies) | P6 Section included on page 6 |
| Confidence in cumulative evidence | 17 | Describe how the strength of the body of evidence will be assessed (such as GRADE) | P5 Confidence in cumulative evidence |

*From: Shamseer L, Moher D, Clarke M, Ghersi D, Liberati A, Petticrew M, Shekelle P, Stewart L, PRISMA-P Group. Preferred reporting items for systematic review and meta-analysis protocols (PRISMA-P) 2015: elaboration and explanation. BMJ. 2015 Jan 2;349(jan02 1):g7647.*
