# Supplementary material for: Thoracic dysfunction in whiplash-associated disorders: a systematic review and meta-analysis protocol
Source: Syst Rev. 2016 Feb 9;5:26. doi: 10.1186/s13643-016-0201-0 (PMC4748634; doi:10.1186/s13643-016-0201-0)
Supplement: Additional file 2: — Medline OvidSP advanced search: 1948-25th November 2015. (DOCX 16 kb) [file 13643_2016_201_MOESM2_ESM.docx]

| 1 | exp Accidents, Traffic/ or exp Whiplash Injuries/ or whiplash associated disorder$.mp. or *Disability Evaluation/ or *Adult/ | 54869 |
| --- | --- | --- |
| 2 | *Pain Measurement/ or *Adult/ or *Whiplash Injuries/ or whiplash.mp. | 13959 |
| 3 | *Adult/ or exp Accidents, Traffic/ or exp Wounds, Nonpenetrating/ or motor vehicle accident.mp. or exp "Wounds and Injuries"/ | 773526 |
| 4 | *Adult/ or Motor vehicle collision.mp. or *"Wounds and Injuries"/ | 49932 |
| 5 | *Thoracic Outlet Syndrome/ or *Whiplash Injuries/ or cervical strain.mp. or *"Sprains and Strains"/ or *Adult/ | 7026 |
| 6 | *"wounds and injuries"/ or *athletic injuries/ or *back injuries/ or *soft tissue injuries/ or *thoracic injuries/ | 76697 |
| 7 | *Thoracic Vertebrae/ or mid-spine.mp. | 9413 |
| 8 | *Kyphosis/ or *"Bone and Bones"/ or dorsal spine.mp. | 48873 |
| 9 | *Musculoskeletal Pain/ or *Musculoskeletal System/ or musculoskeletal.mp. | 36808 |
| 10 | 1 or 2 or 3 or 4 or 5 or 6 | 798153 |
| 11 | 7 or 8 or 9 | 93894 |
| 12 | 10 and 11 | 14607 |
| **13** | limit 12 to (english language and humans and "adult (19 plus years)" and "diagnosis (best balance of sensitivity and specificity)") | **714** |

**Additional File 2**

**Medline OvidSP advanced search: 1948-25^th^ November 2015**
