# Supplementary material for: Thoracic dysfunction in whiplash-associated disorders: a systematic review and meta-analysis protocol
Source: Syst Rev. 2016 Feb 9;5:26. doi: 10.1186/s13643-016-0201-0 (PMC4748634; doi:10.1186/s13643-016-0201-0)
Supplement: Additional file 3: — Example of results/summary table. (DOCX 20 kb) [file 13643_2016_201_MOESM3_ESM.docx]

Additional file 3

Example of results/summary table

| Author | Dysfunction & sample size | Time post injury | Summary results | Quality | | | Comments/study quality |
| --- | --- | --- | --- | --- | --- | --- | --- |
|  |  |  |  | Selection  **** | Comparability  * | Exposure /Outcome  *** |  |
| *Smith et al., 2001* | *Thoracic outlet syndrome*  *N=35* | *2 - 8 months post injury* | *Thoracic outlet syndrome n=30 based on clinical and medical tests*  *Unilateral symptoms n=25*  *Bilateral symptoms n=5* | ** | * | ** | *Sample small & recruitment strategy unclear*  *No a priori power calculation*  *Missing data unaccounted for* |
|  |  |  |  |  |  |  |  |
|  |  |  |  |  |  |  |  |

Note: example only, selection and quality assessment not yet completed
